# Cross-national comparisons of health indicators require standardized definitions and common data sources

**DOI:** 10.1186/s13690-021-00734-w

**Published:** 2021-11-25

**Authors:** Hanna Tolonen, Jaakko Reinikainen, Päivikki Koponen, Hanna Elonheimo, Luigi Palmieri, Mariken J. Tijhuis, for the Joint Action on Health Information (InfAct)

**Affiliations:** 1 Department of Public Health and Welfare, Finnish Institute for Health and Welfare (THL), Helsinki, Finland; 2 Department of Cardiovascular, Endocrine-metabolic Diseases and Ageing, Istituto Superiore di Sanità (ISS), Roma, Italy; 3 National Institute for Public Health and the Environment (RIVM), Bilthoven, the Netherlands

**Keywords:** Health information, Obesity, Hypertension, Diabetes, Asthma, Indicator, Comparability, Standardization

## Abstract

**Background:**

Health indicators are used to monitor the health status and determinants of health of the population and population sub-groups, identify existing or emerging health problems which would require prevention and health promotion activities, help to target health care resources in the most adequate way as well as for evaluation of the success of public health actions both at the national and international level. The quality and validity of the health indicator depends both on available data and used indicator definition. In this study we will evaluate existing knowledge about comparability of different data sources for definition of health indicators, compare how selected health indicators presented in different international databases possibly differ, and finally, present the results from a case study from Finland on comparability of health indicators derived from different data sources at national level.

**Methods:**

For comparisons, four health indicators were selected that were commonly available in international databases and available for the Finnish case study. These were prevalence of obesity, hypertension, diabetes, and asthma in the adult populations. Our evaluation has three parts: 1) a scoping review of the latest literature, 2) comparison of the prevalences presented in different international databases, and 3) a case study using data from Finland.

**Results:**

Literature shows that comparability of estimated outcomes for health indicators using different data sources such as self-reported questionnaire data from surveys, measured data from surveys or data from administrative health registers, varies between indicators. Also, the case study from Finland showed that diseases which require regular health care visits such as diabetes, comparability is high while for health outcomes which can remain asymptomatic for a long time such as hypertension, comparability is lower. In different international health related databases, country specific results differ due to variations in the used data sources but also due to differences in indicator definitions.

**Conclusions:**

Reliable comparison of the health indicators over time and between regions within a country or across the countries requires common indicator definitions, similar data sources and standardized data collection methods.

## Background

Health indicators are used to monitor the health status and determinants of health of the population and population sub-groups and identify existing or emerging health problems which would require prevention and health promotion activities. They also help to target health care resources in the most adequate way as well as for evaluation of the success of public health actions both at the national and international level. Health indicators are also used in international comparisons and benchmarking between countries and estimating changes over time within countries.

For example, at the European level, several health-related indicators are regularly reported in the Health at a Glance reports [1], and the European Core Health Indictor (ECHI) tool [2], both allowing cross-country comparisons and benchmarking of several indicators. More globally, the WHO Global action plan for prevention and control of non-communicable diseases (NCDs) in 2013–2020 has explicitly defined targets related to harmful use of alcohol, physical inactivity, tobacco use, obesity, hypertension, and diabetes, and it promotes monitoring of these indicators [3, 4]. It has also provided definitions for these indicators and recommendations for data sources to ensure comparability of the results between countries.

For cross-country comparisons, standardized definitions of the used indicators are needed. The European Health Examination Survey (EHES) has provided standardized indicator definitions for example for obesity, elevated blood pressure, elevated cholesterol, and diabetes when data from objective health measurements are available [5]. The ECHI shortlist is a more extensive list of standardized indicator definitions including 88 indicators which are split into five main chapters: demographic and socio-economic, health status, health determinants, health services and health promotion. For each indicator, a metadata definition is provided in the documentation sheets [6]. The ECHI indicators cover a wide range of health-related topics, thus they are relevant for several policy areas, supporting the health in all policies (HIAP) approach [7].

Data used to calculate health indicators can be obtained from a variety of data sources such as administrative registers, disease registries, health interview or health examination surveys, and cohort or longitudinal studies [8]. These can be national or regional and the updating frequency may vary by country and data source. One of the common data sources, especially in the international comparisons, is self-reported data from health interview surveys. From another angle, information could also be obtained through objective measurements during the health examination survey or from administrative health records, e.g., covering. in- and out-patient health care visits, and prescribed medications.

In the framework of the Joint Action on Health Information (InfAct – Information for Action), the aim is to create and develop a sustainable solid infrastructure on EU health information through improving the availability of comparable, robust, and policy-relevant data on health status, health determinants and health care services, as well as health system performance information [9]. One specific aim relates to the standardization of health information instruments, tools and methods including health indicators and related data sources.

In this study we will evaluate existing knowledge about comparability of different data sources for the definition of health indicators, compare how selected health indicators presented in different international databases possibly differ, and finally, present the results from a case study from Finland on comparability of health indicators derived from different data sources at national level. Based on these findings we will discuss implications of these potential differences for cross-country comparisons, as well as when estimating time trends at national level.

## Methods

For comparisons, four health indicators, commonly available in international databases and for the Finnish case study, were selected. These indicators were prevalence of obesity, hypertension, diabetes, and asthma in adult population. Our evaluation has three parts: 1) a scoping review of the latest literature, 2) a comparison of the prevalences presented in different international databases, and 3) a case study using data from Finland.

### A scoping review

A scoping review for the comparability of different data sources for estimation prevalence of obesity, hypertension, diabetes, and asthma was conducted focusing to the literature published in English after 2000. For outcomes where limited number of publications were available, also publications from late 1990’s were considered. In comparison to systematic review, a scoping review will provide an overview of the available research evidence answering the question on what kind of information has been presented in the literature [10]. For this scoping review, a search was conducted in December 2020–January 2021 using PubMed and the following search terms in different combinations: obesity**,** hypertension, diabetes, asthma, prevalence differences, different data sources, comparison of different data sources, self-reported, HIS, health examination, health examination survey, medical record, register, administrative data, objective, validation and validity. See more details about the scoping review process in the Appendix 1.

### Review of international health related databases

The following international databases were reviewed for information about the definition and prevalence of obesity, hypertension, diabetes, and asthma: the European Common Health Indicator (ECHI) database through the ECHI data tool [11], the WHO Global Observatory data repository [12], the WHO European Health for All database [13], and the OECD Health data [14].

### Case study from Finland

As a case study for the comparison between different data sources we used data from the Finnish national health examination survey, the National FinHealth Study conducted in 2017 [15]. The FinHealth 2017 survey covered the general population of Finland aged 18+ years and living in the mainland of Finland (i.e., excluding Åland). Data was collected through questionnaires (self-administered and interviewed), physical measurements and collection of biological samples during the visit to the examination centres located around the country. A total of 10,247 persons were randomly selected from the national population register using stratified one- and two-stage sampling and oversampling for person age 80+ years. Participation rate to the health examination was 58% (*n* = 5952). The study was approved by the Ethics Committee of Helsinki and Uusimaa hospital district (37/13/03/00/2016 on 2016-03-22) and written informed consent was obtained from all participants. Informed consent included also consent to link survey data to different administrative registers.

Survey data was linked at the individual level to administrative health registers using the unique personal identification code (PIC) provided for all permanent residents in Finland. This PIC is systematically used in different data sources for identification of individuals.

Linked health registers were Care Register for Health Care [16] and Register of Primary Health Care visits [17] covering all visits for public health care sector but excluding visits to the private health care sector. In both registers, each visit is recorded on individual level together with the date and diagnoses for the visit (ICD-10 or ICPC-2 partly for primary health care visits). These two registers also include information about prescribed medications using ATC (Anatomical Therapeutic Chemical) Classification codes. The Care Register for Health Care also includes information about surgical operations using NCSP (Nordic Classification of Surgical Procedures). For record linkage, permission from the register owners was obtained.

Survey weights were used in the calculation of prevalences in order to take into account the study design and to adjust for non-participation. Cohen’s kappa coefficient (κ) [18] was used to measure the agreement of survey and register data on the individual level.

For estimation of the prevalences from different data sources, definitions given in the Table 1 were used.

**Table 1:** Used definitions of the indicators based on different data sources

Indicator	Survey data		Administrative register data^**a**^
	Self-reported	Measured	
**Obesity**	BMI ≥ 30 based on self-reported height and weight	BMI ≥ 30 based on measured height and weight	ICD-10: E65-E68 ICPC-2: T82 NCSP: JDF00, 01, 10, 11, 20, 21, 32, 96, 97, 98
**Hypertension**	**Yes** to the questions *‘Have you ever been diagnosed for high or elevated blood pressure?’* AND **Yes** to *‘Have you ever used hypertensive medications?’* AND **a response within past 12 months or more recently** for ‘*When was the last time you took medication for high blood pressure?’*	Measured systolic blood pressure ≥ 140 mmHg or measured diastolic blood pressure ≥ 90 mmHg (average of two consecutive measurements) or reported taking medications to lower blood pressure	ICD-10: I10-I13, I15, I67.4, R03.0 ICPC-2: K85-K87
**Diabetes**	**Yes** to type 1 or type 2 diabetes questions *‘Have you ever been diagnosed for diabetes?’* AND **positive response for insulin, tables or their combination** in *‘What prescription medicine do you currently use for diabetes?’*	Fasting glucose ≥7 mmol/l or HbA_1c_ ≥ 48.0 mmol/mol or reported taking medication for diabetes	ICD-10: E10-E14, G59.0, G63.2, H28, G36.0, I79.2, M14.2, M14.6, N08.3 ICPC-2: T89, T90 ATC: A10
**Asthma**	**Yes** to the question *‘Has a doctor ever diagnosed you with asthma?’* AND **within past 12 months or more recently** for the question *´When was the last time you used asthma medication?’*	Spirometry not available	ICD-10: J45, J46 ICPC-2: R96 ATC: R03AK06–08, R03AK10–11, R03AL08–09, R03BA01–02, 05, 07–08, R03DC01, 03

^a^*ICD-10* International Classification of Diseases, 10th revision; *ICPS-2* International Classification of Primary Care, *ATC* Anatomical Therapeutic Chemical; *NCSP* Nordic Classification of Surgical Procedures

## Results

### Scoping review

**Fig. 1:**
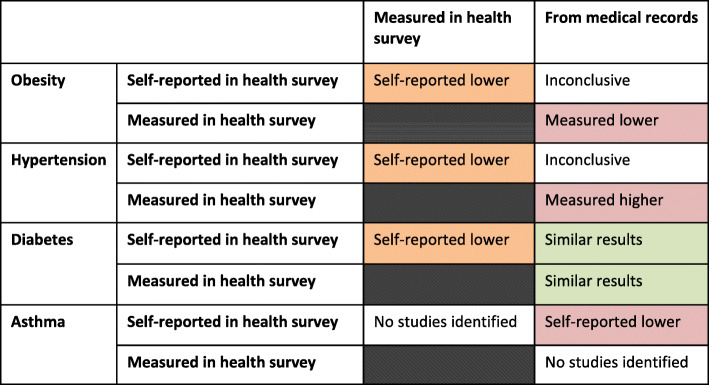
Overall summary of the results from the scoping review about the comparisons of the health outcomes obtained from different data sources

The scoping review focused on differences in the obtained prevalence estimates for obesity, hypertension, diabetes, and asthma when derived from different data sources for same population (Appendix 2). Observed differences between data sources varied by health outcomes (Fig. 1).

For obesity, several studies have demonstrated that prevalence based on self-reported height and weight tends to be lower when compared to prevalence based on measured data, usually from health examination surveys [19–24]. When comparing self-reported results with those obtained from medical registers results are inconclusive, i.e. there is indication that self-reported obesity prevalence would be higher [25] and other studies show similar results between these two sources [32]. Measured obesity prevalences tend to be higher when compared to data from medical records [32].

For hypertension, prevalence based on self-reported information tends results lower prevalence of hypertension in comparison to data obtained through objective survey measurements of blood pressure [21, 26–28]. When results based on self-reported information are compared to register based information, some studies are reporting lower results [26, 29–32], and some higher results [33–36]. For measured hypertension, higher results are often observed in comparison to medical records [29, 32].

For diabetes, in some studies, self-reported information has provided lower diabetes prevalence than prevalence based on objective survey measurements [21, 31] and also self-reported diabetes prevalence has been higher than what has been obtained from medical records [37].

For asthma, limited number of studies comparing the prevalence among adults through different data sources was available even though there were several studies focusing on paediatric asthma. Among adults, reported results indicate that prevalence of asthma based on self-reported information is lower in comparison to medical records [31, 34, 36, 38, 39].

### International health related database

Reviewing four different international databases (ECHI data tool, WHO Global Observatory data repository, WHO Europe Health for All Database, and OECD database) which cover health indicators, we evaluated the definitions used for these indicators, the data sources these indicators were calculated from, and how actual reported prevalences by European Union (EU) Member States (MSs) differed.

Only the ECHI data tool covered all four indicators (obesity, hypertension, diabetes, and asthma), generally results were presented for age group 18+ years, except in the OECD database which presented results for age group 15+ years. Only the ECHI data tool used systematically data from the European Health Interview Survey for all four indicators, i.e. self-reported data. In other databases, data sources varied between indicators but also within indicators between countries (Table 2).

**Table 2:** For different health outcomes, used data sources, coved age group and used indicator definitions in four international health databases

		Database			
		ECHI data tool	WHO Global Health Observatory	WHO European Health for All Database	OECD database
**Data source**		European Health Interview Survey	For obesity and hypertension data from population-based surveys was used, and for diabetes various available data sources were used. Calculations were supplemented with modelling if a country did not have the required data.	For BMI data from the WHO Global Health Observatory, for diabetes data sources vary between countries covering health interview or examination surveys, medical prescriptions, medical reimbursements, diabetes registers, out-patient records, hospital discharge data, and health insurance records individually or in varying combinations.	Sources vary
**Age group covered**		18+	18+	18+	15+
**Indicator**	**Obesity**	BMI ≥ 30 kg/m^2^ based on self-reported data from the European Health Interview Survey	BMI ≥ 30 kg/m^2^ based on measured height and weight	BMI ≥ 30 kg/m^2^ based on measured height and weight	Both self-reported and measured information for combined overweight and obesity BMI ≥ 25 kg/m^2^
	**Hypertension**	Self-reported, having raised blood pressure in past 12 months	Measured systolic blood pressure ≥ 140 mmHg or diastolic blood pressure ≥ 90 mmHg	Not available	Not available
	**Diabetes**	Self-reported, having diabetes in the past 12 months	Raised fasting blood glucose ≥7.0 mmol/l or on medication	ICD-9: 250; ICD-10: E10-E14	Not available
	**Asthma**	Self-reported, having asthma in the past 12 months	Not available	Not available	Not available

**Fig. 2:**
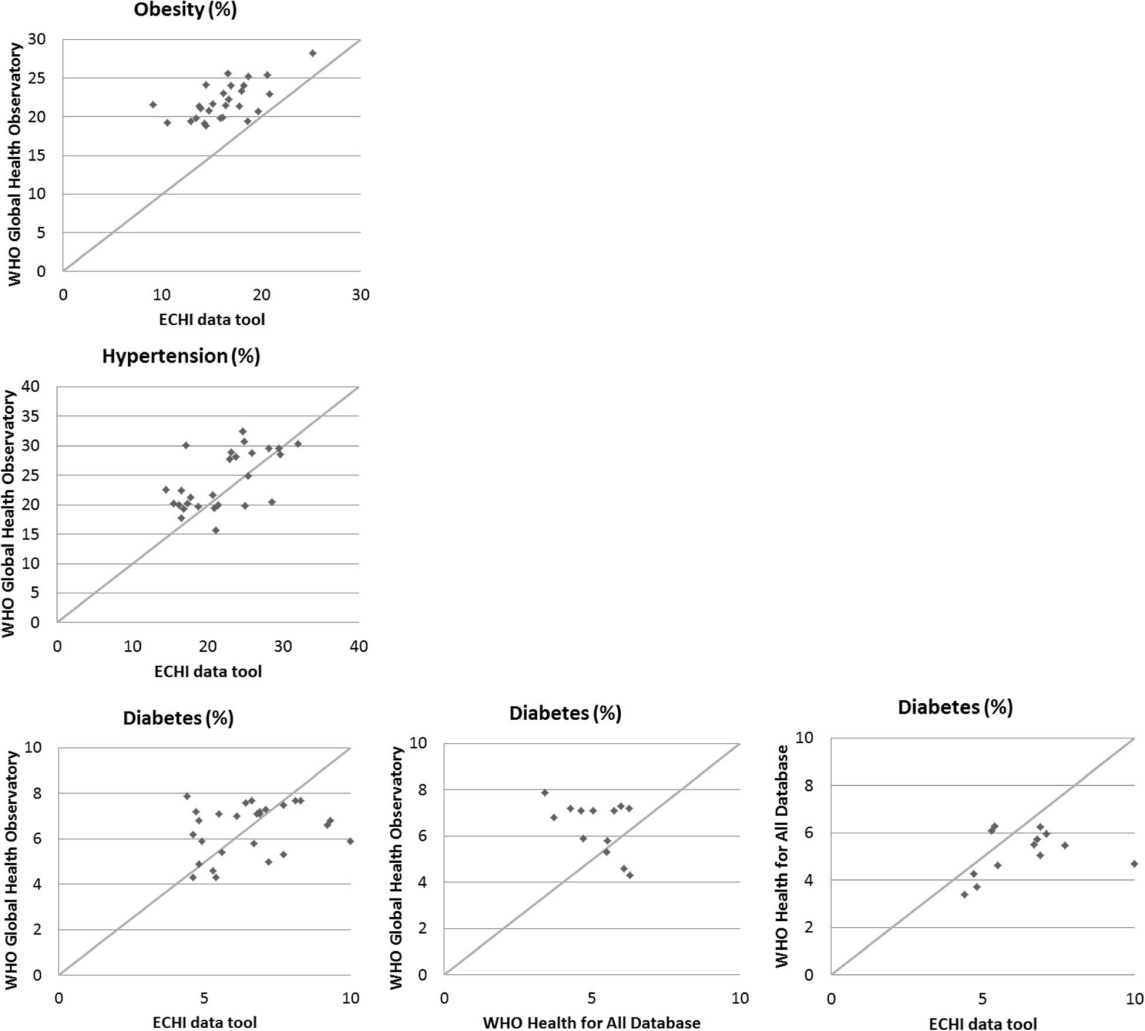
Prevelance (%) of obesity, hypertension and diabetes among EU Member States based data obtained from EHCI data tool (using Eurostat EHIS, wave 2 (2014)), WHO Global Health Observatory for year 2014 and WHO Health for All Database for year 2014, population aged 18+ years

When the actual prevalence estimates at the country level within EU MSs were compared between different international databases, we observed substantial differences (Fig. 2). For comparisons year 2014 was selected since in all databases data for this year was available.

When comparing prevalences presented in different databases (Appendix 2), we observed differences, even though results represent the same age group and year. The OECD data cannot be directly compared with other databases since it covered a different age group and presented prevalence for overweight or obesity instead of obesity only.

For the prevalence of obesity and hypertension, the WHO Global Health Observatory numbers tend to be little higher than those reported in the ECHI data tool which can be explained by different data sources. For diabetes, the difference is more divided.

### Case study from Finland

Obesity, and elevated blood pressure or hypertension, were two indicators which had major differences between data sources. For obesity, prevalence ranged from 2.7% in register-based data to 26.1% when survey data (self-reported or measured) was used, and for hypertension from 16.8 to 45.5%. For diabetes and asthma, the obtained prevalences were rather similar between the data sources (Table 3).

**Table 3:** Prevalences of obesity, high blood pressure, diabetes, and asthma by different data sources in the Finnish case study

Indicator	Data source			
	Self-reported information on survey questionnaire	Objective measurement during the health survey	Self-reported or measured in survey	Register based data
**Obesity**	21.7%	25.4%	26.1%	2.7%
**Elevated blood pressure or hypertension**	24.2%	35.5%	45.5%	16.8%
**Diabetes**	7.1%	6.1%	8.9%	8.5%
**Asthma**	8.9%	–	8.9%	9.5%

Looking at the population level, prevalence of different indicators does provide only one perspective to the comparability of different data sources. It does not tell how much of all the identified cases can be obtained from a specific data source. The agreement of register and survey data in the individual level varied from κ = 0.11 for obesity to κ = 0.83 for diabetes (Table 4)

**Table 4:** Agreement of different health oucomes by different data source in the Finnish case study

Indicator	Total number of identified cases	Cases identified by			Kappa^**a**^
		Only survey data (self-reported and/or measured), n (%)	Only register data, n (%)	Both survey and register data, n (%)	
**Obesity**	1590	1451 (91.3%)	14 (0.1%)	125 (7.9%)	0.11
**Elevated blood pressure or hypertension**	3020	1906 (63.1%)	60 (2.0%)	1054 (34.9%)	0.33
**Diabetes**	652	119 (18.3%)	58 (8.9%)	475 (72.9%)	0.83
**Asthma**	674	120 (17.8%)	157 (23.3%)	397 (58.9%)	0.72

^a^For agreement between register and survey data

.

For obesity, 91% of all cases were identified only by survey data, i.e. those persons did not have any diagnoses in the administrative registers about obesity, 8% were identified as obese by both survey and register, data and only 0.1% had obesity related diagnose but were not identified as obese based on self-reported or measured BMI in the survey.

Also, for hypertension or elevated blood pressure, the majority of the cases were observed in the survey data. Almost 2/3 of all cases were identified only by survey data, 1/3 by both survey and administrative register data, and only few cases (2.0%) were identified only by register data.

For diabetes and asthma, for which at the population level prevalence provided rather similar results between different data sources, we can see that for only 59% of asthma and 73% of diabetes cases were identified by both survey data as well as through administrative registers. For diabetes, most of the additional cases (18%) come from survey data while for asthma, 23% of cases come only from the administrative register data.

## Discussion

Health indicators are widely used for many purposes from monitoring and allocation of available resources to information-based policy decision making. Therefore, it is important to have reliable, well defined indicators which allow at the national level evaluation of trends and differences between regions and population sub-groups. The indicators should allow also cross-country comparisons and benchmarking at the international level.

For reliable health indicators we can define two main components; 1) the data that was used and 2) the definition of the indicator. Both affect the quality of obtained estimates. If your data is biased or incomplete, your indicator estimate will also be biased regardless of the definition you use. On the other hand, if the definition of the indicator is not valid for the phenomena to be measured, high quality data does not help, and your estimates will be biased.

As our study has demonstrated, the same health indicators such as prevalence of obesity, hypertension, diabetes, and asthma, can be defined in several different ways either based on person’s own statement of the situation, using objective measurements, or relying on medical records of health care visits or use of prescribed medications.

From the literature and previous studies, we know that self-reported information may suffer from recall bias [40], awareness bias and from non-response bias [41]. A part of recall bias is something called a telescoping bias [42], where a person perceives recent events as being more remote than they are and distant events as being more recent than they actually are. Awareness bias is evident when a person has never been diagnosed with the health condition for example hypertension which can be asymptomatic for a long time. Non-response bias is well documented for survey research and unfortunate fact is that survey non-response is usually selective and not at random. This may result that those with health problems are not responding to the survey and obtained prevalence estimates get underestimated.

Information obtained through objective health measurements conducted during the health examination survey do not suffer from recall or awareness bias but are still prone for non-response bias similar to self-reported data. Measurements during the survey visit do not correspond to clinical diagnoses. For example, diagnoses of hypertension require subsequent measurements over time. However, measured survey data can be used to identify persons with potential risk and need for further screening.

From medical records, we can obtain information about diagnosed and treated cases but again, the asymptomatic cases which are not yet diagnosed remain uncounted for. There may also be differences in the coverage of the target population by different registers which again may cause some coverage bias to the results. A study from the Netherlands demonstrates relatively good comparability of the data between different registers for diabetes [43]. Combining different registers for example increased the prevalence of diabetes by 3%.

In our case study from Finland, the main limitations are that data from the medical reports does not cover data from occupational health care if provided by private companies the data hasn’t been transferred to the national records, and problems in data transfer from local patient records to the national registers due to differences in the used electronic systems. Also, not all practitioners in the public health centres or out-patient clinics systematically record the diagnoses for each visit. The coverage of hospital data is known to be better.

As has been demonstrated by our results, none of the data sources is perfect but they all have some limitations. In an ideal case, one could combine several different data sources at the individual level and compose indicators based on this combined information. In the Netherlands, different health institutes have formed a consortium which provides information for the Ministry of Health and they jointly define most relevant data sources for specific indicators to be reported [44].

Different international databases mainly rely on publicly available data from different data sources or data specifically provided for them by national authorities. Therefore, availability and comparability of different indicators varies by age group but also by year for which information is available.

When comparing country level prevalence estimates for obesity, hypertension, and diabetes between different international databases, we observed that results from the ECHI data tool were systematically lower than those obtained from WHO Global Health Observatory Database even though they covered the same age group and represented the same year. This can be partly explained by the differences in used data sources. ECHI information is based on self-reported data from the European Health Interview Survey while the WHO Global Health Observatory uses data from objective measurements. Our scoping review also demonstrated that self-reported information tends to result in lower prevalences than measured data.

For many health indicators, standardized definitions are provided by the ECHI shortlist [6]. International organizations such as European Commission (EC), WHO and OECD have worked together towards common definitions. This is an essential step towards comparable data.

Taking into consideration all the pros and cons of different data sources, and through that their effect on indicator definitions, it is important to know what data is used to build the health indicators you use. This is needed to ensure that when you compare results between countries or over time, you use same data sources, same definitions, and same reference age group. Even when the data sources are similar, there may be methodological differences which limit data comparability, e.g. used sampling frames and response rates in EHIS, using interviews or self-administered mailed questionnaires, as well as differences in patient record systems, clinical and recording practices affecting register data.

### Strengths and limitations of the study

Strengths of our study are that we have evaluated possible effects of indicator definitions and used data sources using three different methods: a scoping review, review of existing international databases and conducting a case study on existing data from Finland.

We focused only in four health indicators in this study, and this is obviously also a limitation since some other indicators might have provided different results. Also, our case study covered only one country and results may not be directly generalizable to other countries due to differences in the available data sources. But it was not possible to obtain information for other countries covering all three data sources; self-reported, objective measurements and register data, on same individuals.

## Conclusions

Without common definition of the health indicators and standardized methods for data collection, such as provided by ECHI, EHIS or EHES, cross-country comparisons are not meaningful as it is not possible to determine whether observed differences are due to real differences between the countries or to different definitions of the same indicators. It is essential to ensure that similar data sources and standardized data collections are used in these cross-country comparisons. As it is almost impossible to fully standardize data collection, the cross-country comparisons should be made with caution keeping in mind the possible sources of bias.

## Figures and Tables

**Fig. 3:**
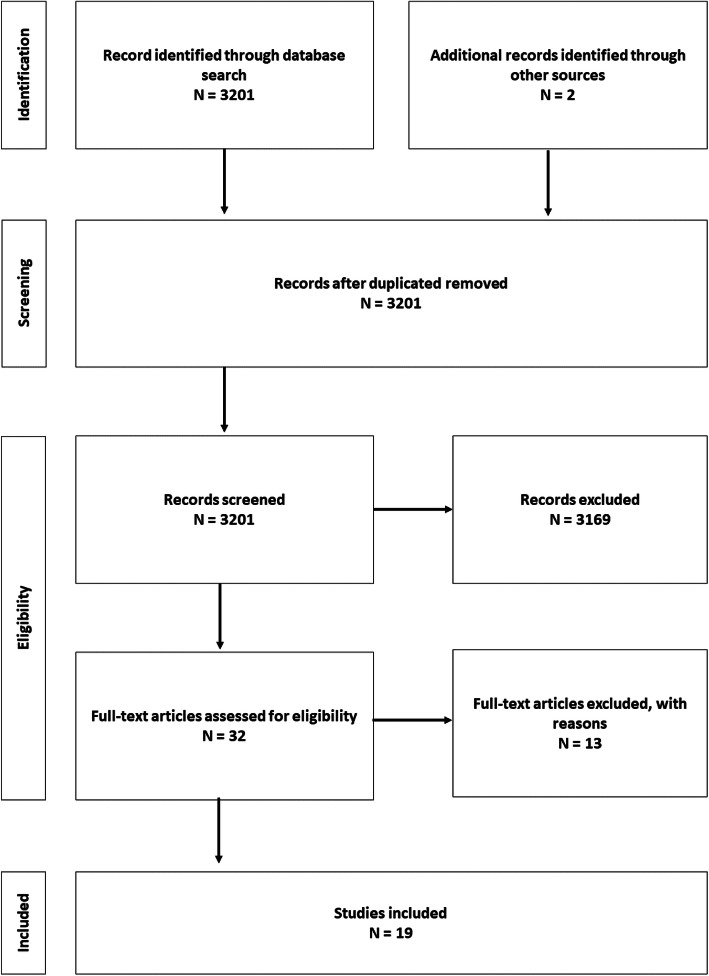
PRISMA chart for the scoping review

## Data Availability

The FinHealth 2017 survey data are not publicly available due to restrictions based in the General Data Protection Regulation (GDPR) on sensitive data such as personal health data. The access to the data may be requested through the Finnish Institute for Health and Welfare (THL) Biobank (https://thl.fi/en/web/thl-biobank/for-researchers).

## Appendix 2

**Table 5:** Results of the scoping review

Outcome	Study:	Study aims:	Results:
Obesity	Maukonen et al., 2018 [21]	Target group: adults Comparison: self-reported vs. measured Type of study: review of 62 studies	Self-reported in comparison to measured: • height was overestimated ⇧ • weight was underestimated ⇩ • BMI was underestimated ⇩ • prevalence of overweight was underestimated ⇩ from 1.8%-points to 9.8%-points • prevalence of obesity was underestimated ⇩ from 0.7%-points to 13.4%-points Bias was greater among overweight and obese persons in comparison to normal weight persons.
	Luke et al., 2017 [25]	Target group: adults Comparison: measured vs. medical records Type of study: cross-sectional comparison	Measured in comparison to medical records • obesity rate was underestimated ⇩ Of the 16 different obesity class, ethnicity, and sex strata compared between EHR and NHANES patients, 14 (87.5%) included similar obesity estimates (i.e., overlapping 95% CIs).
	Yun et al., 2006 [23]	Target group: self-reported vs. measured Type of study: cross-sectional comparison	Self-reported in comparison to measured: • prevalence of overweight was underestimated ⇩ • prevalence of obesity was underestimated ⇩ Underestimation was prevalent across different gender, race, age and education groups
	Paccaud et al., 2001 [24]	Target group: adults Comparison: measured vs.self-reported Type of study: cross-sectional comparison	Self-reported in comparison to measured: • weight was underestimated ⇩ • height was overestimated ⇧ • BMI was underestimated⇩ • prevalence of obesity was underestimated ⇩ The differences larger for women than for men.
Hypertension	Atwood et al., 2013 [29]	Target group: adults Comparison: self-reported vs measured or electronic health records (HER) Type of study: cross-sectional comparison	Self-reported in comparison to measured: • prevalence of hypertension was slightly underestimated ⇩ Self-reported in comparison to electronic health records (HER): • prevalence of hypertension was underestimated ⇩ Measured in comparison to electronic health records (EHR): • prevalence of hypertension was slightly underestimated ⇩
	Goncalves et al., 2018 [27]	Target group: adults Comparison: self-reported hypertension (HTN) vs. clinical diagnosis Type of study: a systematic review and meta-analysis of 22 studies	Self-reported in comparison to medical diagnoses: • In most studies prevalence on hypertension was underestimated ⇩
	Peng et al.; 2016 [30]	Target group: adults Comparison: measured vs. medical record Type of study: cross-sectional comparison	Measured in comparison to medical records: • prevalence of hypertension was overestimated ⇧
	Frank, 2016 [31]	Target group: adults Comparison: self-reported vs. measured or medical records Type of study: cross-sectional comparison	Self-reported in comparison to measured: • prevalence of hypertension was underestimated ⇩ Self-reported in comparison to medical records: • prevalence of hypertension was underestimated ⇩ Measured in comparison to medical records: • prevalence of hypertension was similar ⇔
Diabetes	Aguilar-Palacio et al., 2014 [37]	Target group: adults Comparison: self-reported vs. electronic medical records (EMR) Type of study: cross-sectional comparison	Self-reported in comparison with electronic medical records: • prevalence of diabetes was overestimated ⇧
Asthma	Mukherjee et al., 2016 [38]	Target group: General population, all ages Comparison: self-reported vs. medical records Type of study: cross-sectional comparison	Self-reported in comparison to medical records: • Prevalence of asthma was overestimated ⇧
	Huzel et al., 2002 [39]	Target group: adults Comparison: self-reported vs. medical records Type of study: cross-sectional comparison	Self-reported in comparison to medical records: • Prevalence of asthma attack in past 12 months was overestimated ⇧
Obesity, Hypertension, Diabetes	Zellweger et al., 2014 [33]	Target group: adolescents, adults and elderly Comparison: self-reported vs. measurement or electronic health record Type of study: cross-sectional comparison	Self-reported in comparison to measured: • prevalence of obesity was overestimated ⇧ • prevalence of hypertension was overestimated ⇧ • prevalence of diabetes was similar ⇔ Self-reported in comparison to medical records: • prevalence of obesity was similar ⇔ • prevalence of hypertension was overestimated ⇧ • prevalence of diabetes was similar ⇔ Measured in comparison to medical records: • prevalence of obesity was overestimated ⇧ • prevalence of hypertension was overestimated ⇧ • prevalence of diabetes was similar ⇔
	Schenker et al., 2010 [22]	Target group: adults Comparison: self-reported vs. measured Type of study: cross-sectional comparison	Self-reported in comparison to measured: • prevalence of obesity was underestimated ⇩ • prevalence of hypertension was underestimated ⇩ • prevalence of diabetes was slightly underestimated ⇩
Diabetes, Hypertension, Asthma	Lujic et al., 2017 [35]	Target group: adults Comparison: self-report vs. medical records Type of study: cross-sectional comparison	Self-reported in comparison to medical records: • Prevalence of hypertension was overestimated ⇧ • Prevalence of diabetes was overestimated ⇧ • Prevalence of asthma was overestimated ⇧
	Violán et al., 2013 [32]	Target group: adults Comparison: self-reported vs. electronic health records (EHR) Type of study: cross-sectional comparison	Self-reported in comparison to electronic medical records: • prevalence of hypertension was slightly underestimated ⇩ • prevalence of diabetes was underestimated ⇩ • prevalence of asthma was overestimated ⇧
Diabestes, Hypertension	Paalanen et al., 2018 [26]	Target group: adults Comparison: self-reported vs. measured or medical records (MRs) Type of study: a literature review of 30 studies	Self-reported in comparison to measured: • prevalence of hypertension was underestimated ⇩ from −15.9%-points to + 3.9%-points • prevalence of diabetes was underestimated ⇩ from −2.9%-points to + 0.9%-points Self-reported in comparison to medical records: • prevalence of hypertension was underestimated ⇩ from − 14.9%-points to + 19.7%-points • prevalence of diabetes was underestimated ⇩ from − 3.0%-points to + 0.9%-points
	Robinson et al., 1997 [36]	Target group: adults Comparison: self-reported vs. medical records Type of study: cross-sectional comparison	Self-reported in comparison to medical records: • prevalence of hypertension was overestimated ⇧ • prevalence of diabetes was underestimated ⇩
	Barber et al., 2010 [34]	Target group: adults Comparison: self-report vs. medical records Type of study: cross-sectional comparison	Self-reported in comparison to medical records: • prevalence of hypertension was overestimated ⇧ • prevalence of diabetes was similar ⇔
Diabetes, Asthma	Berete et al., 2020 [45]: Comparing health insurance data and health interview survey data for ascertaining chronic disease prevalence in Belgium. *Arch Public Health* 78:120. doi: 10.1186/s13690-020-00500-4	Target group: adolescents and adults Comparison: self-reported vs. insurance data Type of study: cross-sectional comparison	Self-reported in comparison to insurance data: • prevalence of diabetes was similar ⇔ • prevalence of asthma was overestimated ⇧

## Appendix 3

**Table 6:** Prevalences of obesity, hypertension, diabetes and asthma presented in different international databases for 2014

Country	Obesity					Hypertension		Diabetes			Asthma
	ECHI	WHO GHO	WHO HFA	OECD - self-reported^**a**^	OECD – measured^**a**^	ECHI	WHO GHO	ECHI	WHO GHO	WHO HFA	ECHI
Austria	14,3	19,2	19,2	46,7		21,1	15,6	4,9	5,9		4,4
Belgium	13,7	21,4	21,4		51	16,5	17,8	5,3	4,6	6,08	4,3
Bulgaria	14,4	24,1	24,1	55,4		29,6	28,6	6,4	7,6		2,7
Croatia	18	23,4	23,4			24,6	32,4	7,1	7,3	5,98	3
Cyprus	13,9	21,1	21,1			17,3	20,2	6,1	7		4,3
Czech Republic	18,7	25,2	25,2			23,7	28,1	7,7	7,5		4,5
Denmark	14,4	18,9	18,9			17,7	21,2	4,6	4,3		6,5
Estonia	19,7	20,7	20,7	52	51,3	22,9	27,7	5,5	7,1	4,62	3,1
Finland	17,8	21,4	21,4	52,1		24,9	19,8	7,7	5,3	5,49	9,2
France	14,7	20,8	20,8	46,1		14,4	22,5	10	5,9	4,71	8,8
Germany	16,4	21,5	21,5	50,7		28,5	20,5	7,2	5		6,1
Greece	16,9	24	24	56,2		20,9	19,5	9,2	6,6		4,4
Hungary	20,6	25,4	25,4	54,5	62,3	31,9	30,4	8,1	7,7		4,9
Ireland	18,2	24	24			15,5	20,2	4,6	6,2		9
Italy	10,5	19,3	19,3	46,4		20,6	21,7	6,7	5,8	5,52	4,8
Latvia	20,8	23	23	52	54,6	29,4	29,6	4,7	7,2	4,26	3,5
Lithuania	16,6	25,6	25,6	53,3		28,1	29,6	4,4	7,9	3,41	2,7
Luxembourg	15,1	21,7	21,7	48	58,1	16,5	22,4	5,6	5,4		6,8
Malta	25,2	28,2	28,2			21,4	19,9	8,3	7,7		5,8
Netherlands	12,9	19,5	19,5	48,2		16,8	19,3	5,4	4,3	6,28	5,5
Poland	16,7	22,3	22,3	53,3		23,1	29	6,6	7,7		4,1
Portugal	16,1	19,9	19,9	52,8		25,3	24,9	9,3	6,8		5
Romania	9,1	21,6	21,6			17,1	30,1	4,8	6,8	3,72	2
Slovakia	15,9	19,8	19,8	54,2		25,8	28,8	6,9	7,2	6,26	3,9
Slovenia	18,6	19,5	19,5	55,6		24,8	30,7	6,9	7,1	5,04	5
Spain	16,2	23,1	23,1	51,5		18,7	19,7	6,8	7,1	5,74	4,5
Sweden	13,4	19,8	19,8	46,5		16,2	19,9	4,8	4,9		7,9

^a^For overweigh or obesity

## Abbreviations

ATC: Anatomic Therapeutic Chemical
EC: European Commission
ECHI: European Core Health Indicator
EHES: European Health Examination Survey
EHIS: European Health Interview Survey
HIS: Health Interview Survey
ICD-10: International Classification of Disease, 10th revision
ICPC-2: International Classification of Primary Care
InfAct: Joint Action on Health Information
NCD: Non-communicable diseases
WHO: World Health Organization
